# Gut microbiota impact on lung diseases: a mini review of clinical evidence

**DOI:** 10.1128/iai.00430-25

**Published:** 2026-03-24

**Authors:** Chuanfeng Liu, Lingying Dan, Xuebin Wang, Lingyong Chen, Xingxing Yuan

**Affiliations:** 1 Department of Pulmonary and Critical Care Medicine, Lishui Hospital of Traditional Chinese Medicine, Lishui, China; 2 Department of Endocrinology, Lishui Hospital of Traditional Chinese Medicine, Lishui, China; 3 First Clinical Medical College, Heilongjiang University of Chinese Medicine 118437, Harbin, China; University of California at Santa Cruz, Santa Cruz, California, USA

**Keywords:** gut microbiota, gut-lung axis, metabolites, clinical significance, dysbiosis

## Abstract

The gut-lung axis represents a bidirectional communication network through which the gut microbiota (GM) influences respiratory health. This mini-review synthesizes clinical evidence on the role of the GM in lung diseases. We focused exclusively on human clinical trials, randomized controlled trials, meta-analyses, and systematic reviews, sourced from major databases after duplicate removal. The evidence indicates that GM dysbiosis is a significant risk factor for the susceptibility and severity of various respiratory conditions, including asthma, chronic obstructive pulmonary disease (COPD), cystic fibrosis (CF), and infections, such as COVID-19 and pneumonia. Specific microbial signatures and metabolic profiles, particularly involving short-chain fatty acids (SCFAs), are associated with disease states and outcomes. Interventions like probiotics, prebiotics, synbiotics, and fecal microbiota transplantation (FMT) show promise in modulating the GM and improving clinical parameters, though their efficacy can be inconsistent and influenced by confounding factors. In conclusion, the GM is a promising therapeutic target for lung diseases. However, future research must prioritize large-scale, longitudinal clinical trials and deeper mechanistic investigations to establish causality and develop effective, personalized microbiome-based therapies.

## INTRODUCTION

The field of microbiome research has been transformed by advanced technologies, such as shotgun metagenomic sequencing and sophisticated data analytics, providing unprecedented resolution of the gut microbiota (GM) and its functions. This has intensified interest in how host–microbe interactions influence human health, particularly through systemic pathways like the “gut-lung axis.” This axis describes a bidirectional communication network in which the GM and its metabolites exert influence on the health of the distant pulmonary system (1). A growing body of evidence implicates GM dysbiosis in the pathogenesis and progression of major chronic respiratory diseases, including chronic obstructive pulmonary disease (COPD), cystic fibrosis (CF), idiopathic pulmonary fibrosis (IPF), and asthma (2, 3). The connection between the gut and lungs is complemented by the oral-lung axis. The proximity and shared connection of the oral cavity and lungs via the upper respiratory tract establish a crucial link. Evidence indicates that the oral microbiome can serve as a reservoir that influences the composition of the lung microbiome (4). This relationship underscores their collective impact on respiratory health and suggests that oral hygiene interventions could potentially mitigate the risk of pulmonary diseases, such as pneumonia, by reducing the translocation of pathogenic bacteria into the lower airways (5).

The GM influences host physiology through multiple mechanisms. Beneficial gut symbionts metabolize dietary components to produce essential metabolites like short-chain fatty acids (SCFAs), which help neutralize toxins and regulate the function of distant organs, thereby maintaining systemic homeostasis (6). Furthermore, the GM is a fundamental modulator of the host immune system. It stimulates local intestinal immune responses, including the production of secretory immunoglobulin A, which helps contain microbes within the gut. This local priming subsequently influences systemic immunity, helping to shape adaptive immune responses and potentially enhancing the host’s resistance to respiratory infections (7). Diet is a primary modifiable factor that shapes GM composition and function, directly impacting the gut-lung axis. For example, high consumption of ultra-processed foods, typically low in fiber and high in additives, is associated with dysbiosis, increased systemic inflammation, and elevated risk of cardio-metabolic disorders (8). Consequently, dietary patterns represent a significant public health concern and a critical area for research on GM-mediated lung health.

Given the GM’s central role, therapeutic strategies aimed at modulating its composition are being explored for pulmonary conditions. Fecal microbiota transplantation (FMT), probiotics, and prebiotics have demonstrated anti-inflammatory potential (9). Studies suggest they can ameliorate asthma pathogenesis and may improve outcomes in severe respiratory infections like COVID-19 (10, 11). While FMT is established for recurrent *Clostridioides difficile* infection, its application in respiratory diseases requires further investigation regarding safety and efficacy (12). Synbiotics, which combine probiotics and prebiotics, offer a targeted approach and have shown safety and effectiveness in managing specific pulmonary infections (13). Probiotics and prebiotics have also been associated with reduced symptom severity and inflammation in COVID-19 patients, likely by reinforcing gut barrier integrity and modulating immune responses (14).

The COVID-19 pandemic itself has highlighted the GM’s role, as SARS-CoV-2 infection can disrupt both the respiratory and gut microbiomes, potentially contributing to long-term sequelae (15). Nutritional supplements also show potential; for instance, vitamin D supplementation demonstrates a protective effect against asthma development and exacerbations, possibly mediated through immune regulation and GM interactions (16). However, the field faces substantial challenges. The pulmonary microbiome is highly heterogeneous, making it difficult to establish universal links between specific microbial signatures and disease parameters (17). Establishing definitive cause-and-effect relationships remains a hurdle, as current evidence often cannot conclusively prove that GM alterations cause specific respiratory outcomes (18, 19). Furthermore, while environmental factors like air pollution can alter respiratory microbial communities, robust evidence directly linking these shifts to detrimental host metabolic changes is still lacking (20).

A critical limitation is that commonly used metrics like alpha-diversity often fail to capture the full complexity of the GM-lung health relationship. Although reduced alpha-diversity is a hallmark of conditions like CF, it is an insufficient standalone predictor for many other pulmonary diseases (21). This underscores the need for more extensive, well-designed clinical trials focused on GM modulation in chronic respiratory conditions. Such studies are essential to move beyond correlation and elucidate the precise molecular and immunological mechanisms governing the gut-lung axis. This mini-review synthesizes clinical evidence on the GM’s impact on lung diseases. It highlights the potential of GM modulation as a therapeutic strategy while acknowledging the current limitations posed by methodological inconsistencies, confounding factors, and the challenge of proving causation. A concerted effort toward rigorous trials and deeper mechanistic understanding is required to translate the promise of the gut-lung axis into effective clinical applications.

## IMMUNOLOGICAL MECHANISMS OF THE GUT-LUNG AXIS

The gut-lung axis is largely mediated by immunological pathways, through which GM-derived signals educate and modulate the host immune system, thereby influencing respiratory immunity. A key mechanism involves the priming of immune cells in the gut-associated lymphoid tissue, which then migrate to the lung mucosa via the common mucosal immune system. GM-derived metabolites, particularly SCFAs like acetate, propionate, and butyrate, play a pivotal role in systemic immune regulation. SCFAs inhibit histone deacetylases, promoting a tolerogenic environment by expanding regulatory T cells (Tregs) and suppressing pro-inflammatory T-helper 17 (Th17) cell responses (22). This SCFA-mediated Treg/Th17 balance is crucial for controlling excessive inflammation in conditions such as asthma and COPD. Furthermore, SCFAs influence the function of antigen-presenting cells, such as dendritic cells and alveolar macrophages, shifting them toward an anti-inflammatory phenotype and enhancing phagocytic activity against respiratory pathogens (23).

The GM also shapes innate lymphoid cell (ILC) populations in the lungs. Group 3 innate lymphoid cells (ILC3s), which are important for barrier immunity and tissue repair, can be influenced by GM signals. Conversely, dysbiosis may promote group 2 innate lymphoid cells (ILC2s), which drive type 2 inflammation in allergic asthma (24). B cell function and immunoglobulin production are also under GM influence. Gut-derived signals can promote the class-switching and production of IgA, which, upon translocation to the respiratory tract, can neutralize pathogens and maintain mucosal homeostasis (25). Pattern-recognition receptors, including toll-like receptors (TLRs) and NOD-like receptors, recognize microbial-associated molecular patterns from the GM. This interaction continuously tunes the basal tone of the systemic immune system, preparing it to respond appropriately to lung insults. For instance, GM-dependent TLR activation is essential for optimal antiviral interferon responses, linking gut microbial health to resistance against viral respiratory infections like influenza and SARS-CoV-2 (26). The GM exerts its effects on the lungs through a complex network of immune mechanisms, involving metabolic signaling, cellular trafficking, and receptor-mediated crosstalk. Understanding these pathways is essential for developing targeted interventions that leverage the gut-lung axis.

## GUT MICROBIOTA COMPOSITION AS A RISK FACTOR FOR LUNG INFECTIONS

The composition of the GM is increasingly recognized as a critical factor influencing susceptibility to and severity of lung infections and chronic respiratory diseases. Specific microbial signatures, characterized by the abundance or depletion of particular taxa, are emerging as significant risk factors. For instance, distinct GM profiles are associated with asthma. *Ruminococcaceae* UCG-014 and *Barnesiella* show positive correlations with asthma, whereas *Candidatus Soleaferrea* shows a protective, negative correlation. In adults, reduced levels of *Akkermansia*, a genus associated with mucosal health, serve as a biomarker for asthma onset. Childhood-onset asthma is linked to an increased abundance of *Collinsella* and *Ruminococcaceae* UCG-014, alongside reduced levels of the *Family XIII AD3011* group, *Eisenbergiella*, and *Ruminiclostridium 6* (27).

The COVID-19 pandemic has further underscored this link. The abundance of *Actinomyces oris* and *Mycoplasma* correlates with increased COVID-19 mortality, suggesting their potential use in risk stratification (28, 29). Even recovered patients often exhibit persistent GM dysbiosis (30). Characteristic shifts include a reduction in beneficial SCFA-producing bacteria (*Faecalibacterium*, *Eubacterium*, *Roseburia*, *Lachnospira*) and overall *Firmicutes*, accompanied by an increase in potentially pathogenic taxa, such as *Bacteroides*, *Clostridium*, *Eggerthella*, and *Rothia*. A lower *Firmicutes*/*Bacteroidetes* ratio is also common (31, 32). Specific markers like gut *Ruminococcus*, *Escherichia*, and *Candida* abundance are associated with COVID-19 infection, differentiating it from community-acquired pneumonia, which is prognostically linked to *Staphylococcus* and *Candida* (33).

Dysbiosis is also a feature of chronic lung diseases. In CF, patients exhibit reduced GM diversity, depletion of SCFA producers, and enrichment of bacterial populations associated with inflammation (34, 35). Pulmonary arterial hypertension is linked to alterations, including increased *Eubacterium fissicatena* and *Lachnospiraceae CG-004* (36). In lung cancer patients, GM profiles differ significantly, with *Escherichia*, *Enterococcus*, and *Lactobacillus* emerging as potential biomarkers for early diagnosis (37). In COPD and pulmonary tuberculosis, airway microbiota analysis reveals distinct beta diversity patterns, characterized by reduced *Streptococcus* and *Prevotella* and increased *Haemophilus* and *Pseudomonas* (38, 39). IPF severity is influenced by GM composition; *Bifidobacteriaceae* and *Ruminococcaceae* exert protective effects, while an abundance of *Coprococcus 2* is associated with more severe symptoms (40).

**Fig 1:**
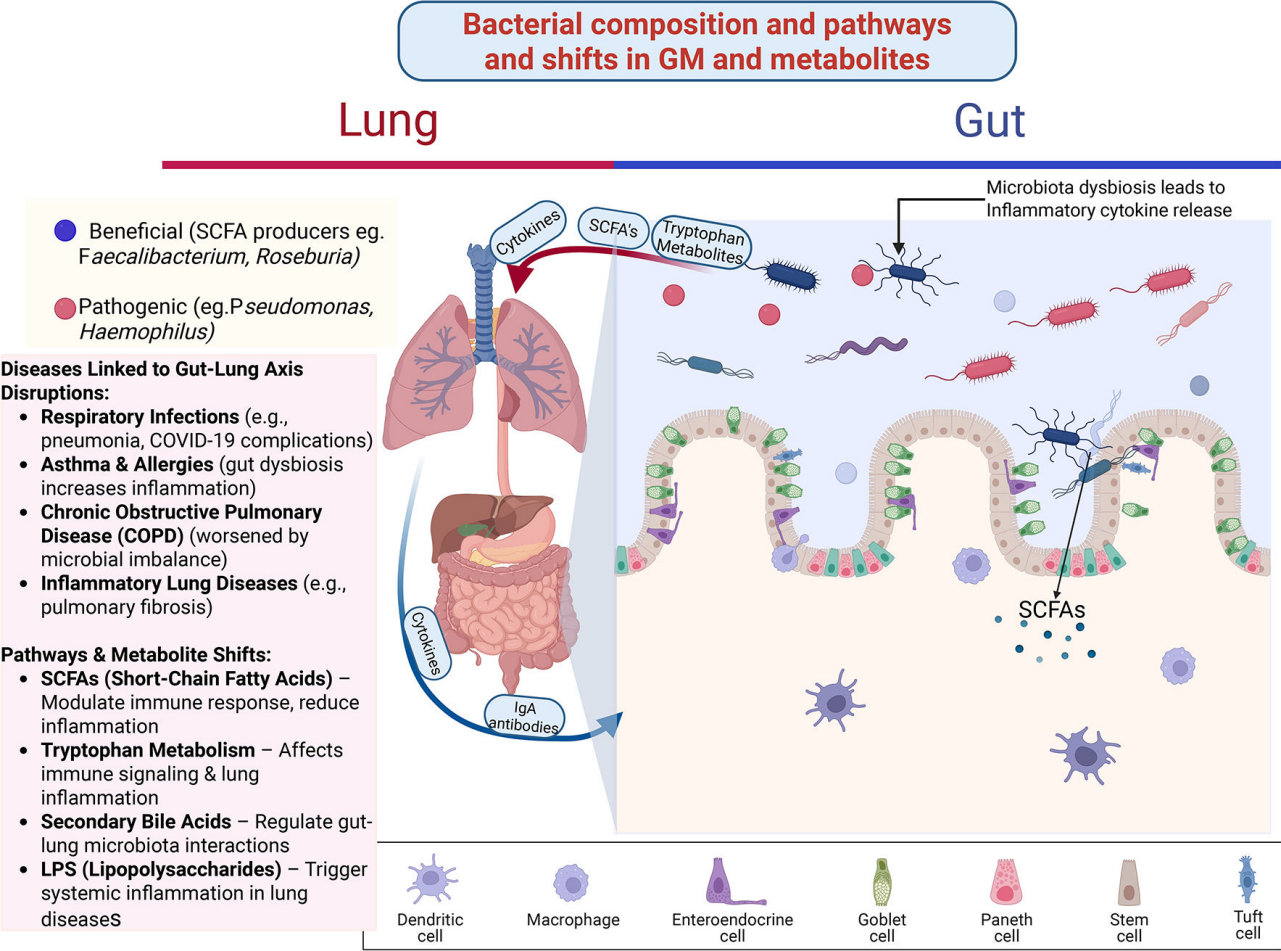
The gut-lung axis illustrates how alterations in GM composition and metabolite production influence pulmonary immunity and respiratory disease outcomes. The right panel shows the gut environment, where dysbiosis, characterized by reduced beneficial SCFA-producing bacteria (e.g., *Faecalibacterium*, *Roseburia*) and increased pathogenic taxa, triggers inflammatory cytokine release and disrupts microbial metabolite profiles. Key metabolites affected include SCFAs, tryptophan-derived metabolites, bile acids, and lipopolysaccharides (LPS), each of which modulates immune signaling and systemic inflammation. These gut-derived metabolites and cytokines enter circulation and affect the lung (left panel), where they modulate local immune responses, IgA-mediated defenses, and macrophage activity. Dysregulated gut-lung communication contributes to multiple pulmonary conditions, including respiratory infections (e.g., pneumonia, COVID-19), asthma and allergic inflammation, COPD, and inflammatory lung diseases, such as pulmonary fibrosis. The figure highlights the bidirectional flow of immune signals and metabolites linking gut dysbiosis to lung inflammatory pathways and disease severity.

Environmental factors like urbanization are associated with GM alterations, such as reduced diversity and depletion of beneficial genera like *Bifidobacterium*, which correlate with increased allergy susceptibility (41). Childhood respiratory diseases are frequently associated with low GM alpha-diversity and characteristic shifts involving *Ruminococcus* and *Bifidobacterium* (42). The GM profile serves as a significant risk modifier for lung health. Specific constellations of bacteria are consistently associated with susceptibility and outcomes across a spectrum of respiratory conditions. However, harnessing this knowledge requires larger, longitudinal studies to establish robust causal links. Figure 1 below depicts a visual illustration of the bacterial composition and pathway shifts in the gut-lung axis.

## PATHWAYS AND SHIFTS IN GM AND METABOLITES

The gut-lung axis is mediated by microbial metabolites, immune modulation, and potential microbial translocation, influencing a spectrum of respiratory conditions. A key illustration is acute respiratory distress syndrome (ARDS), where dysbiosis in the lung microbiota correlates with elevated alveolar TNF-α, a driver of inflammation (43). The presence of gut-specific bacteria, such as *Bacteroides* spp., in the bronchoalveolar lavage fluid of ARDS patients suggests a breakdown in gut-lung barriers and active bidirectional communication (44).

In asthma, co-administration of the probiotic Probio-M8 with the inhaler Symbicort Turbuhaler has shown promise in regulating airway nitric oxide levels and improving symptom control (45, 46). This intervention enriches beneficial SGBs like *Bifidobacterium longum* and *Bifidobacterium animalis*, while reducing pathogenic bacteria (47). This restructuring shifts the host metabolome, increasing levels of beneficial metabolites like linoleoyl ethanolamide and erythronic acid, demonstrating how probiotics can reshape the metabolic landscape to promote health. In non-small cell lung cancer (NSCLC), concurrent chemoradiotherapy induces dysbiosis, typified by an increase in *Proteobacteria* and a reduction in *Firmicutes* (48). Kyoto Encyclopedia of Genes and Genomes (KEGG) pathway analysis has linked GM composition to progression-free survival (PFS), with enriched pathways for arginine and fatty acid biosynthesis associated with PFS, underscoring the GM’s role in treatment efficacy (49). Furthermore, immunotherapy in NSCLC patients was associated with an increase in beneficial butyrate-producing bacteria like *Agathobacter*, suggesting potential biomarkers for treatment response (50).

The gut-lung axis also manifests in pediatric diseases. In infants with CF, bile acids in bronchoalveolar lavage fluid shape the airway inflammatory and microbial landscape (51). Supplementation with *Lactobacillus rhamnosus* GG in children with CF fostered a *Bifidobacteria*-dominant profile, correlating with reduced pulmonary exacerbations and improved lung function (52). In children with community-acquired pneumonia (CAP), the development of antibiotic-associated diarrhea (AAD) is linked to microbial features, including an increased abundance of *Lachnospiraceae* (53, 54). Combining zinc with the probiotic mixture *Bifico* was effective against diarrhea, characterized by increased *Bifidobacterium* and decreased *Escherichia coli* populations (55). In tuberculosis, *Lactobacillus casei* supplementation demonstrated hepatoprotective effects, reducing cholestasis-related liver indices and markers of enterocyte damage, suggesting improved gut barrier function (56).

**Fig 2:**
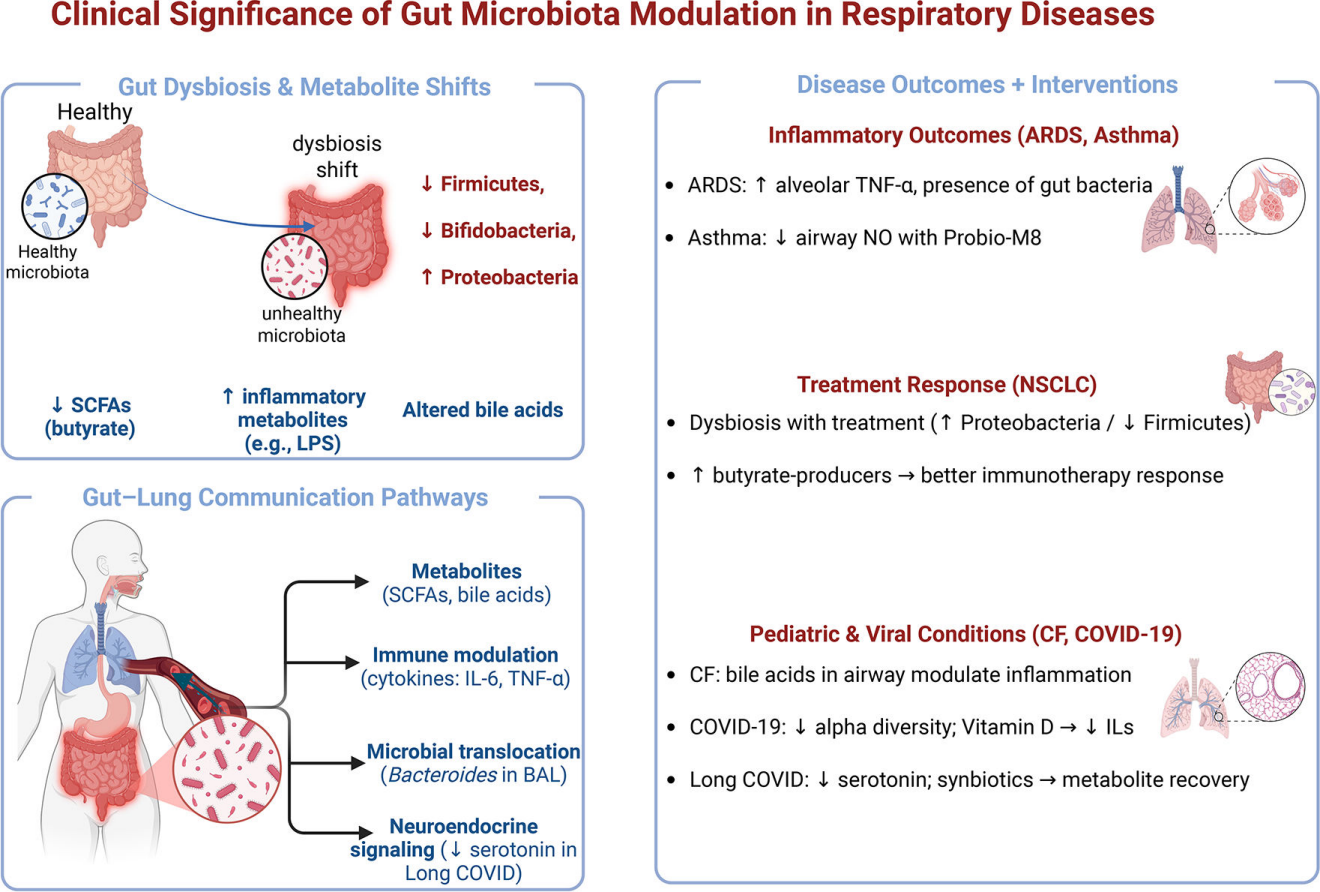
Gut dysbiosis leads to characteristic shifts in microbial composition, including reduced *Firmicutes* and *Bifidobacteria* and increased *Proteobacteria*, that together alter key metabolites, such as SCFAs, LPS, and bile acids. These metabolite and microbial changes influence the gut-lung axis through four major communication routes: (i) metabolic signaling (e.g., altered SCFAs and bile acids), (ii) immune modulation via cytokines, such as IL-6 and TNF-α, (iii) microbial translocation evidenced by the presence of gut-associated bacteria in bronchoalveolar lavage fluid, and (iv) neuroendocrine effects, including reduced serotonin levels observed in long COVID. These pathways contribute to distinct respiratory disease outcomes. In inflammatory conditions, such as ARDS and asthma, dysbiosis is associated with elevated alveolar TNF-α, presence of gut-derived bacteria, and reduced airway nitric oxide following Probio-M8 supplementation. In NSCLC, treatment-associated dysbiosis (↑ *Proteobacteria*, ↓ *Firmicutes*) and enrichment of butyrate-producing taxa correlate with improved immunotherapy responses. In pediatric and viral diseases, bile acids shape airway inflammation in CF, while COVID-19 pathology is linked to reduced microbial diversity, vitamin D-associated suppression of inflammatory interleukins, and serotonin depletion contributing to long COVID symptoms. Synbiotic supplementation demonstrates potential for restoring beneficial metabolites in post-acute disease.

COVID-19 has provided further insights. SARS-CoV-2 infection can trigger significant GM dysbiosis, with lower alpha diversity associated with more severe disease (57). GM-mediated reduction in serotonin (5-HT) production is proposed as a mechanism contributing to neuropsychiatric and gastrointestinal symptoms in long COVID (58). Synbiotic interventions may ameliorate fatigue, with specific mixtures increasing metabolites like choline and creatine (59). Dietary interventions, such as beetroot juice, increased beneficial butyrate-producing genera in long COVID patients (60, 61). Vitamin D3 supplementation in acute COVID-19 reduced inflammatory interleukins and was associated with increased trimethylamine N-oxide (TMAO), correlating with less severe infection and shorter hospital stays (62, 63). However, probiotic efficacy is nuanced; they may not consistently restore severely depleted beneficial taxa or alter the fecal metabolome, indicating that microbial population changes do not always translate to functional metabolic alterations (64). The pathways connecting GM to respiratory health are complex and bidirectional. Dysbiosis and resultant metabolite shifts influence inflammation, immunity, and disease progression across numerous conditions. An overview of the clinical and mechanistic significance of GM modulation in respiratory diseases is depicted in Fig. 2 below.

## CLINICAL SIGNIFICANCE OF GUT MICROBIOTA ON LUNG DISEASES

Translating knowledge of the gut-lung axis into clinical applications is supported by a growing body of human evidence demonstrating the significance of GM modulation. Direct modification of the GM is gaining traction. A clinical trial showed that a mixture of *Leonurus japonicus Houtt* and *Lactiplantibacillus plantarum KC3* is safe and shows therapeutic potential for chronic respiratory diseases (65). In COPD, distinct GM signatures are characterized by increased *Lachnospiraceae* and downregulated starch metabolism pathways, largely driven by smoking (66). Critically, FMT has demonstrated efficacy in alleviating core COPD pathologies, including reducing alveolar destruction and improving lung function (67). Furthermore, conventional therapies like Erythromycin can modulate the microbiome, reducing the burden of *Burkholderia* while increasing beneficial *Veillonella* and *Prevotella* (68).

## RESEARCH DURING THE COVID-19 PANDEMIC YIELDED SIGNIFICANT CLINICAL INSIGHTS

Acute phase modulation: specific probiotics suppressed nasopharyngeal SARS-CoV-2 viral load and reduced lung infiltrates, an effect correlated with increased specific IgG and IgM antibodies, suggesting an immune-mediated mechanism (69). Combating long COVID: the synbiotic preparation SIM01 significantly alleviated symptoms of post-acute COVID-19 syndrome (70). FMT has also emerged as a strategy for ameliorating persistent long COVID symptoms, particularly in patients with diarrhea (71). Vulnerable populations: the microbiome formula SIM01 reversed adverse health outcomes in elderly diabetic patients with COVID-19, highlighting the importance of microbiome health in high-risk groups (72). The GM’s influence extends to lung cancer outcomes. Evidence suggests that modulating the GM could enhance the efficacy of immunotherapy for malignancies like mesothelioma and NSCLC, potentially improving responses to immune checkpoint inhibitors (49). Emerging clinical evidence is compelling. Specific GM signatures are linked to disease states, and interventions including probiotics, synbiotics, and FMT demonstrate measurable benefits, from improving lung function in COPD to modulating immune responses in COVID-19. This underscores the GM as a legitimate therapeutic target with significant clinical relevance (Table 1).

**TABLE 1:** Summary of clinical evidence linking gut microbiota to lung diseases^*a*^

Category	Dysbiosis features	Key metabolites/pathways	Clinical significance and intervention evidence
Asthm**a**	Increased: *Ruminococcaceae UCG-014, Barnesiella, Collinsella* Decreased: *Candidatus Soleaferrea, Akkermansia,* Family XIII AD3011 group	SCFA (acetate, propionate, butyrate) deficiency Shift in overall metabolome (linoleoyl ethanolamide)	Specific microbial signatures are associated with disease susceptibility and onset Probio-M8 + inhalers can improve symptom control and enrich beneficial bifidobacteria Soluble fiber improves symptoms, though not always via expected SCFA changes
COPD	Reduced diversity Increased: *Lachnospiraceae* Altered metabolic pathways (downregulated starch metabolism)	TMAO associated with long-term mortality	Dysbiosis is largely driven by smoking Fecal microbiota transplantation (FMT) shows efficacy in reducing alveolar destruction and improving lung function in models
CF	Reduced alpha-diversity Depletion of SCFA producers (*Faecalibacterium*) Enrichment of pro-inflammatory populations	Bile acids in airways shape inflammation	*L. rhamnosus* GG can foster a *Bifidobacterium*-dominant profile, correlating with reduced exacerbations Vitamin D supplementation benefits remain unclear
COVID-19	Reduced alpha-diversity Decreased: SCFA producers (*Faecalibacterium, Lachnospira*) Increased: *Bacteroides, Clostridium, Candida*	Reduced 5-HT in long COVID Increased TMAO post-Vitamin D3	Specific probiotics can reduce viral load and lung infiltrates SIM01 and FMT alleviate long COVID symptoms GM profile is distinct from other pneumonias and useful for risk stratification
NSCLC	Potential biomarkers: *Escherichia, Enterococcus, Lactobacillus* Therapy-induced dysbiosis (↑ *Proteobacteria*, ↓ *Firmicutes*)	KEGG pathways for arginine/fatty acid biosynthesis linked to progression-free survival	GM composition influences immunotherapy efficacy (↑ *Agathobacter* with response) Modulating GM is a potential adjuvant to enhance chemotherapy and immunotherapy
Pneumonia and infections	CAP linked to *Staphylococcus* and *Candida* AAD in children linked to ↑ *Lachnospiraceae*		Synbiotics may reduce ventilator-associated pneumonia risk, though efficacy can be confounded *Bifico* + zinc is effective against AAD
IPF	Protective: *Bifidobacteriaceae, Ruminococcaceae* Associated with severity: *Coprococcus 2*		GM composition influences disease severity, suggesting a modifiable risk factor

^*a*^ COPD, chronic obstructive pulmonary disease; NSCLC, non-small cell lung cancer; CAP, community-acquired pneumonia; AAD, antibiotic-associated diarrhea; SCFAs, short-chain fatty acids; TMAO, trimethylamine N-oxide; FMT, fecal microbiota transplantation; KEGG, Kyoto Encyclopedia of Genes and Genomes.

## LIMITATIONS OF GUT MICROBIOTA EFFECTS ON LUNG DISEASES

Despite the compelling framework of the gut-lung axis, translating GM insights into effective clinical interventions faces significant limitations. It is premature to expect GM-targeted strategies to have a broad impact during acute respiratory pandemics, with clinical significance often observed only in specific subpopulations, such as individuals with comorbidities like obesity (73). Confounding factors are a major challenge. For instance, while prophylactic synbiotics may reduce ventilator-associated pneumonia, their efficacy can be obscured by concurrent critical conditions like sepsis (74). Similarly, early-life *Lactobacillus* supplementation can temporarily modify the infant GM but has not consistently translated into lasting asthma prevention, highlighting a disconnect between transient GM shifts and durable disease modification (75). This pattern extends to other interventions, such as vitamin D supplementation in CF, where anticipated benefits remain elusive (76). The influence of GM metabolites is complicated by host factors. In exacerbated COPD, the GM-dependent metabolite TMAO is associated with long-term mortality, but this relationship is heavily modulated by the patient’s age and comorbidities (77). Furthermore, societal-level factors often exert a stronger influence than GM modulation alone (78). Mechanistic understanding also faces hurdles, as some protective effects cannot be fully explained by observed dysbiosis patterns (79). In lung cancer, regulating the intestinal microbiome as an adjuvant to chemotherapy demands a careful balance of the intestinal-pulmonary microecology and must demonstrably improve patient quality of life (80). Research is further complicated by variables like tumor type, smoking history, and nutritional status (81).

**Fig 3:**
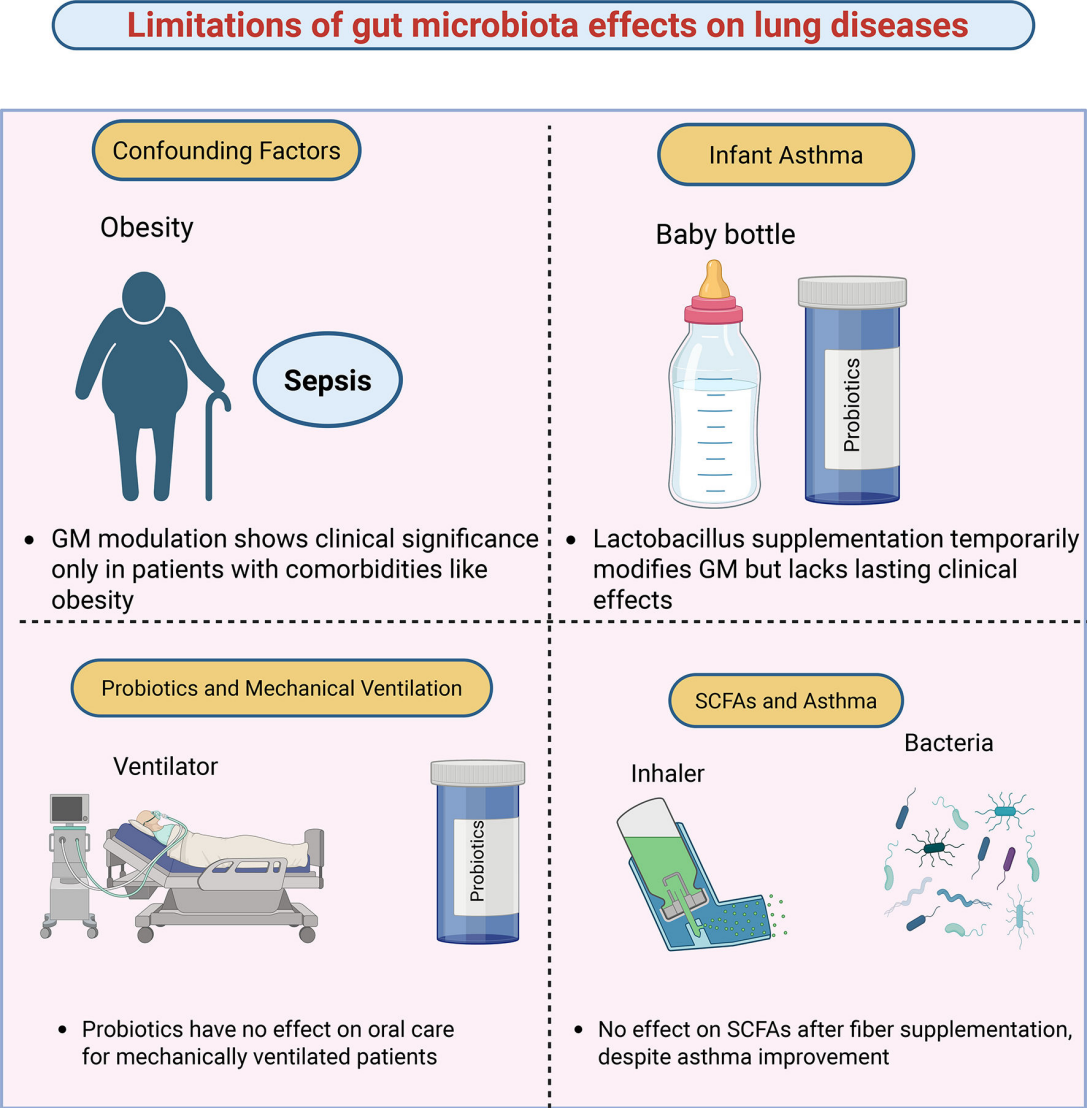
Key limitations of GM modulation in influencing lung diseases. (Top left) Clinical significance is often observed only in specific subpopulations, such as individuals with comorbidities like obesity. (Top right) In infant asthma, *Lactobacillus*-based probiotic supplementation can transiently alter gut microbiota composition but does not produce sustained clinical improvement. (Bottom left) Among mechanically ventilated patients, probiotic administration shows no impact on oral care outcomes or lung-related complications, indicating limited utility in critical care settings. (Bottom right) Despite clinical improvement in asthma symptoms following dietary fiber supplementation, no corresponding increase in SCFAs is observed, highlighting a disconnect between microbiota-modifying strategies and measurable metabolite shifts. Collectively, these limitations emphasize that GM modulation does not uniformly translate into respiratory benefits and may be constrained by patient-specific, clinical, and metabolic factors.

A recurring theme is the disconnect between GM composition, metabolites, and clinical endpoints. Soluble fiber supplementation improves asthma symptoms yet may not produce expected changes in SCFAs (19). Integrative analyses suggest that improvements in childhood asthma are more strongly linked to shifts in the overall metabolome rather than specific GM changes (82). Similarly, probiotic interventions in CF patients sometimes shift GM composition but show no significant effect on crucial clinical parameters like pulmonary exacerbations (83). Finally, probiotics have proven ineffective as an alternative to chlorhexidine for preventing ventilator-associated complications (84). In summary, while biologically plausible, the clinical impact of GM modulation is often modest, transient, or obscured by confounding factors. Moving forward requires research designs that account for host factors, environmental influences, and complex microbial interactions to develop interventions with robust clinical effects. Figure 3 illustrates the major limitations and challenges in gut-lung axis research.

## CONCLUSION

The exploration of the gut-lung axis represents a paradigm shift in our understanding of respiratory health, moving the focus from a localized organ system to a complex, interconnected network where the GM exerts profound systemic influence. This review synthesizes compelling evidence that GM dysbiosis, a state of microbial imbalance characterized by reduced diversity, depletion of beneficial commensals like SCFA-producing *Faecalibacterium*, and enrichment of opportunistic pathogens, is intricately linked to the pathogenesis, susceptibility, and severity of a wide spectrum of respiratory conditions. These range from chronic inflammatory diseases, such as asthma, COPD, and idiopathic pulmonary fibrosis, to acute infections, including influenza, bacterial pneumonia, and notably, COVID-19. The identification of specific microbial signatures, such as reduced *Akkermansia* in asthma or increased *Actinomyces oris* associated with COVID-19 mortality, underscores their emerging potential as diagnostic and prognostic biomarkers. The pathways mediating this cross-talk are multifaceted, primarily involving microbial metabolites like SCFAs and TMAO that systemically modulate host immunity, the education and trafficking of immune cells via the common mucosal immune system, and the direct translocation of microbes or their components across compromised barriers.

Therapeutic modulation of the GM holds significant, albeit complex, promise. Interventions, such as FMT, have demonstrated efficacy in restoring microbial balance and have shown potential in alleviating COPD pathology and persistent long COVID symptoms. Probiotics, prebiotics, and synbiotics exhibit beneficial effects across various contexts; specific strains can modulate immune responses and reduce viral load in acute COVID-19, ameliorate asthma control, reduce pulmonary exacerbations in cystic fibrosis, and improve outcomes in childhood pneumonia. Furthermore, dietary interventions, particularly the reduction of ultra-processed foods and increased intake of dietary fiber, stand as crucial, modifiable factors that fundamentally shape GM composition and its systemic inflammatory tone.

However, translating this exciting potential into robust, widely applicable clinical practice faces substantial limitations. A primary challenge is establishing definitive causality; observed associations between GM dysbiosis and disease do not always prove the GM is the primary driver. The field is also hampered by significant heterogeneity in lung and gut microbiome composition between individuals, in host immune responses, and in study methodologies, which complicates comparisons and meta-analyses. Crucially, simple ecological metrics like alpha-diversity often fail to capture the functional complexity of the GM-lung relationship. Clinical trials frequently reveal a troubling disconnect: interventions may successfully alter GM composition without inducing changes in key metabolic pathways, or they may yield measurable microbial shifts without translating into consistent, significant clinical benefits for patients.

Therefore, harnessing the full therapeutic potential of the gut-lung axis demands a concerted, multi-faceted research effort. Future directions must prioritize several key areas: (i) the implementation of large-scale, longitudinal, and rigorously controlled clinical trials specifically focused on chronic respiratory diseases; (ii) a deeper mechanistic exploration that moves beyond correlations to elucidate precise causal pathways using germ-free animal models and advanced *in vitro* systems; (iii) the development of sophisticated analytical frameworks that integrate multi-omics data (genomics, metabolomics, proteomics) to move beyond simple diversity metrics toward a holistic understanding of microbial community function; (iv) the standardization of sampling, sequencing, and analytical methodologies across research centers to enable meaningful data synthesis; and (v) the adoption of personalized medicine approaches that account for individual host genetics, baseline microbiome, comorbidities, and environmental context. Only through such dedicated and collaborative efforts can we truly translate the profound insights of the gut-lung axis into effective, safe, and personalized microbiome-targeting strategies for the prevention and treatment of debilitating respiratory diseases worldwide.
